# Macro Monte Carlo dose calculation for very high energy electron (VHEE) radiotherapy

**DOI:** 10.1002/mp.70539

**Published:** 2026-06-22

**Authors:** Chengchen Zhu, Florian Amstutz, Claire M. Costantini, Werner Volken, Marco F. M. Stampanoni, Peter Manser, Michael K. Fix

**Affiliations:** ^1^ Division of Medical Radiation Physics and Department of Radiation Oncology Inselspital, Bern University Hospital, and University of Bern Bern Switzerland; ^2^ Department of Physics University of Zurich Zurich Switzerland; ^3^ Institute for Biomedical Engineering ETH Zurich and PSI Villigen Switzerland

**Keywords:** dose calculation, Macro Monte Carlo, radiotherapy, very high energy electron (VHEE)

## Abstract

**Background:**

Very high energy electron (VHEE) radiotherapy has gained growing interest owing to its potential to reach deep‐seated targets and induce FLASH effect. Dose calculations can be performed using analytical or Monte Carlo (MC) methods. Analytical approaches enable rapid dose computation but suffer from limited accuracy in heterogeneous media, whereas MC methods provide high accuracy at the expense of substantial computational cost. Macro Monte Carlo (MMC) is a local‐to‐global method designed to improve dose calculation efficiency compared to general‐purpose MC methods. In MMC, particle transport is based on precalculated transport data generated with general‐purpose MC simulations on specific geometries, which is subsequently used to model particle transport over macroscopic steps within the absorber, avoiding computationally expensive microscopic tracking. MMC made it to a standard electron dose calculation engine in a commercial treatment planning system. However, to date, MMC has not been investigated for electron energies above 25 MeV.

**Purpose:**

To develop and validate an MMC framework for VHEE radiotherapy that improves dose calculation efficiency while preserving accuracy compared to general‐purpose MC methods for electron energies up to 250 MeV.

**Methods:**

Local simulations were performed using EGSnrc with monoenergetic electron pencil beams incident perpendicularly on spherical geometries (0.2‐25 MeV) with radii of 0.5‐3 mm, and slab geometries (25‐250 MeV) of 2 mm thickness, composed of various materials. Physical quantities including energy loss, lateral displacement, and angular distributions of primary and secondary particles were scored and stored in a database. This database was subsequently used to transport electrons step‐by‐step in the global simulations, employing slab‐based transport at energies ≥25 MeV and switching to spherical geometries for electron energies <25 MeV to account for increased scattering. Energy deposition was scored in a 3D dose grid. MMC dose calculations were validated against EGSnrc for monoenergetic VHEE beams (50‐250 MeV) incident on homogeneous and heterogeneous slab phantoms, using pencil beams, parallel spot beams with 1 mm radius, and parallel beams with a field size of 5 × 5 cm^2^. MMC and EGSnrc dose calculations were also performed for two patient CT datasets. Comparisons between MMC and EGSnrc were conducted using integrated depth dose curves, lateral dose profiles, and 3D gamma analysis with 2%/1 mm and 2%/2 mm (global) criteria and a 10% dose threshold. All simulations were performed with statistical uncertainties below 1%, and computation times were recorded.

**Results:**

Integrated depth dose curves and lateral dose profiles agreed within 2% of the maximum dose for all cases considered. For homogeneous and heterogeneous phantoms, MMC dose distributions yielded gamma passing rates above 97% (2%/1 mm) and 99% (2%/2 mm), respectively, compared to EGSnrc. For patient CT datasets, gamma passing rates exceeded 94% (2%/1 mm) and 97% (2%/2 mm). Overall, MMC achieved up to a 27‐fold improvement in dose calculation efficiency compared to EGSnrc.

**Conclusions:**

An MMC framework for VHEE dose calculation was successfully developed and validated for electron energies up to 250 MeV. The method demonstrated good agreement with EGSnrc while providing up to an order‐of‐magnitude improvement in dose calculation efficiency for the studied cases.

## 1 INTRODUCTION

Conventional radiotherapy treatments predominantly employ photon beams to deliver high doses to tumors, to eradicate malignant cells. Compared with photons, charged particles offer the advantage of a finite penetration depth, which can reduce dose deposition in healthy tissues distal to the tumor. Among clinically used charged particles, electrons are inexpensive to accelerate compared with heavier particles such as protons, as they can be produced using relatively compact linear accelerators, whereas proton therapy requires larger accelerator facilities. Conventional linear accelerator based electron therapy uses electron energies between 4 and 22 MeV and is therefore mainly restricted to the treatment of superficial targets due to their limited range of only a few centimeters.^1^

In recent years, there has been increasing interest in the use of very high energy electrons (VHEE), typically defined as electrons with energies between 50 and 250 MeV, for radiotherapy applications.^2^ This interest is driven by several advantageous physical characteristics of VHEE beams: (1) increased range and reduced lateral scattering, leading to sharper penumbrae compared with conventional electron beams;^3^, ^4^ (2) suitability for ultra‐high dose rate (UHDR) delivery, with the potential to induce the FLASH effect in deep‐seated body locations;^5^, ^6^, ^7^ (3) the ability to magnetically confine, steer, or focus the beam,^8^, ^9^ and (4) low sensitivity to tissue heterogeneities.^10^

The potential of VHEE radiotherapy has motivated several studies on VHEE treatment planning and dose delivery.^9^, ^11^, ^12^, ^13^, ^14^ The practical applicability of these approaches is, however, partly constrained by the accuracy and computational efficiency of the underlying dose calculation algorithms. Ronga et al. proposed an analytical dose calculation method for VHEE beams based on Fermi‐Eyges theory, enabling highly efficient computation of dose distributions.^15^ This model was subsequently implemented in the open‐source matRad treatment planning system and validated for bone and lung heterogeneities, achieving passing rates above 90% using a 2D gamma analysis with 2%/1 mm criteria.^16^

While analytical methods provide fast dose calculations, Monte Carlo (MC) algorithms are generally regarded as more accurate, particularly in complex scenarios such as heterogeneous patient geometries derived from CT imaging, as they explicitly model the underlying particle interaction physics.^17^, ^18^ General‐purpose MC codes, including e.g. EGSnrc,^19^ Geant4,^20^ and Penelope,^21^ have been used to compute dose distributions from VHEE beams and often serve as reference standards in the absence of experimental data.^3^, ^12^ However, the high level of accuracy provided by MC simulations is associated with substantial computational cost, motivating the development of more efficient MC‐based dose calculation techniques that preserve accuracy.

To address this challenge, variance reduction methods have been introduced in radiotherapy dose calculations. One such approach is the macro Monte Carlo (MMC) method, which has been successfully applied to electron^22^, ^23^, ^24^ and proton beam dose calculations.^25^, ^26^ These studies reported efficiency gains of approximately one order of magnitude for electrons and up to two orders of magnitude for protons compared with benchmark general‐purpose MC codes, namely EGSnrc for electrons and Geant4 for protons. In the MMC framework, detailed local MC simulations are first performed in predefined geometries, such as spheres or slabs of various sizes and materials, using a range of monoenergetic incident particles (local simulations). Physical properties of the particles from the local particle transport are stored in a database. During the macroscopic transport (global simulation), particle parameters are sampled from this database at each step, defining the particle track in the global simulation, which is performed through a sequence of macro steps. Energy deposition is scored within a 3D dose grid, yielding the dose distribution.

To date, MMC dose calculation for electrons has been implemented and validated only for energies up to 25 MeV, using spheres as local geometries.^23^, ^24^ No MMC implementations have yet been developed for VHEE beams with energies up to 250 MeV. In this work, we develop an MMC method tailored for VHEE dose calculations and validate its performance against EGSnrc using both academic phantoms and patient CT datasets. A more efficient and accurate dose calculation algorithm compared to general‐purpose MC methods can accelerate the overall treatment planning process, enabling more extensive exploration of the VHEE modality and facilitating studies on larger patient cohorts.

## 2 METHODS

In this work, EGSnrc (version 2020) was employed both for the local precalculations and as reference MC code for validation. Default EGSnrc transport settings were used, with Rayleigh scattering disabled (negligible in the considered energy range^19^) and the impulse approximation applied for Compton scattering. Electrons and positrons were transported down to a total energy cutoff (ecut) of 0.6 MeV, while photons were transported down to 0.1 MeV (pcut). The local and global simulations are described in the following sections.

### 2.1 Local simulations

Local precalculations consisted of general‐purpose MC simulations of monoenergetic electron transport in predefined geometries. For electron energies up to 25 MeV, electron transport was modeled using the database and transport developed by Fix et al., where spherical (“Kugel”) geometries of varying radii and materials were used to generate the database.^23^, ^24^ At higher energies of 25–250 MeV, electrons exhibit reduced lateral scattering; therefore, infinitely wide slab geometries were adopted for the local simulations. The slab thickness represents a trade‐off between accuracy and computational efficiency: larger slabs improve efficiency by reducing the number of macro steps in the global transport but may introduce excessive approximations that degrade dose calculation accuracy, whereas thinner slabs yield less approximations and hence more accurate dose calculation at the cost of increased computational effort. In this first study on MMC for VHEE, slab geometries with a thickness of 2 mm were chosen as a practical starting point, as it is expected to yield accurate dose calculations for voxel sizes commonly used in radiotherapy. The slabs were composed of materials corresponding to those used for the spherical geometries in Fix et al.^23^, ^24^ Depending on the material, between 10^7^ and 5∙10^7^ primary electrons were simulated to obtain statistical uncertainties of the data in the database below 0.5%.

Particle tracks and interactions were simulated using general‐purpose MC simulations and investigated to characterize electron transport in slab geometries for electron energies above 25 MeV. In these simulations, the electrons were transported via multiple Coulomb scattering steps and could undergo Møller scattering or Bremsstrahlung interactions, producing secondary particles. Depending on the energy and momentum transferred in these interactions, secondary particles may have lower (shorter range) or higher energies (longer range), resulting in a local or non‐local energy deposition, respectively. Based on this, different “channels” were defined and separated during the local simulations, in which relevant physical quantities were scored and stored in a database.

**FIGURE 1:**
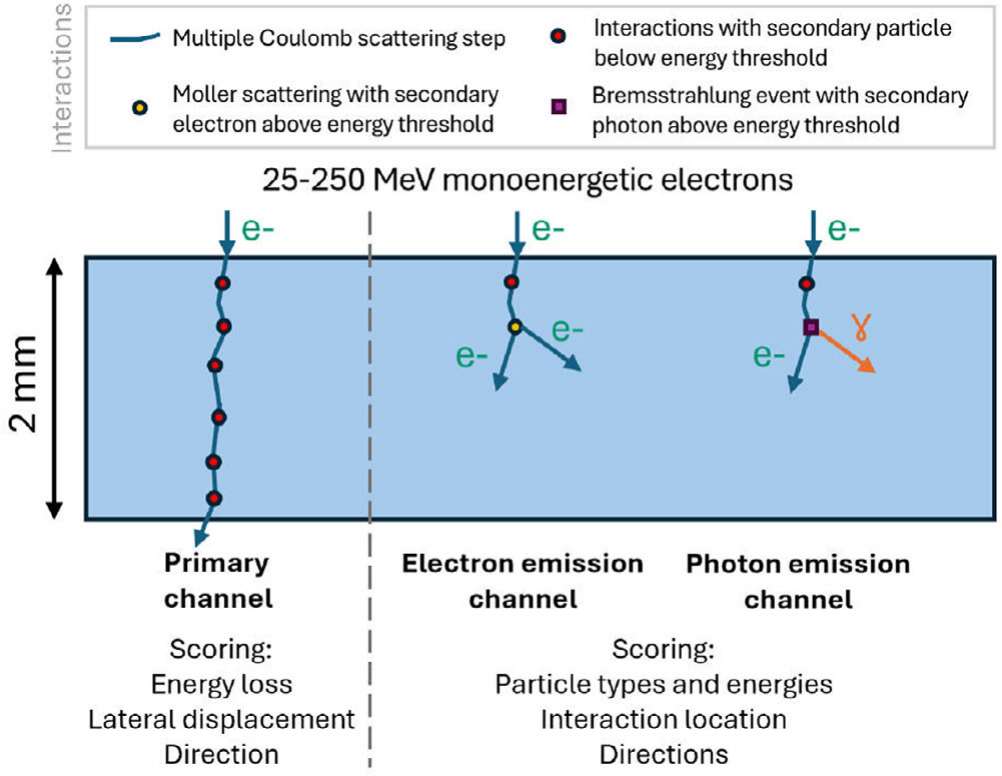
Schematic illustration of the local simulations of monoenergetic electron beams impinging on a 2 mm slab geometry. Multiple Coulomb scattering steps are shown as solid blue lines. Møller or Bremsstrahlung interactions producing secondary particles below the energy thresholds are included in the primary channel (left), whereas interactions producing higher energy electrons or photons are classified into the electron emission channel (middle) and photon emission channel (right), respectively. Simulations were performed using 25–250 MeV electrons in different materials, with channel‐specific physical quantities scored.

Energy thresholds of 0.6 MeV (total energy) for electrons and 0.1 MeV for photons were introduced to distinguish between local and non‐local energy deposition; these thresholds coincide with the EGSnrc transport cutoffs (ecut and pcut), respectively. If the incoming electron experienced only multiple Coulomb scattering within a slab, or if Møller scattering or Bremsstrahlung produced only secondary particles below the corresponding energy thresholds, the exiting electron's energy loss, lateral displacement, and angular distribution were scored (*primary channel*). Conversely, if a Møller scattering or Bremsstrahlung event produced secondary particles with energies above the thresholds, the interaction location, as well as the energy and direction of all particles immediately after the interaction were explicitly recorded (*electron emission* and *photon emission channels*). A schematic overview of the local simulations and the recorded quantities is shown in Figure 1.

### 2.2 Global simulations

**FIGURE 2:**
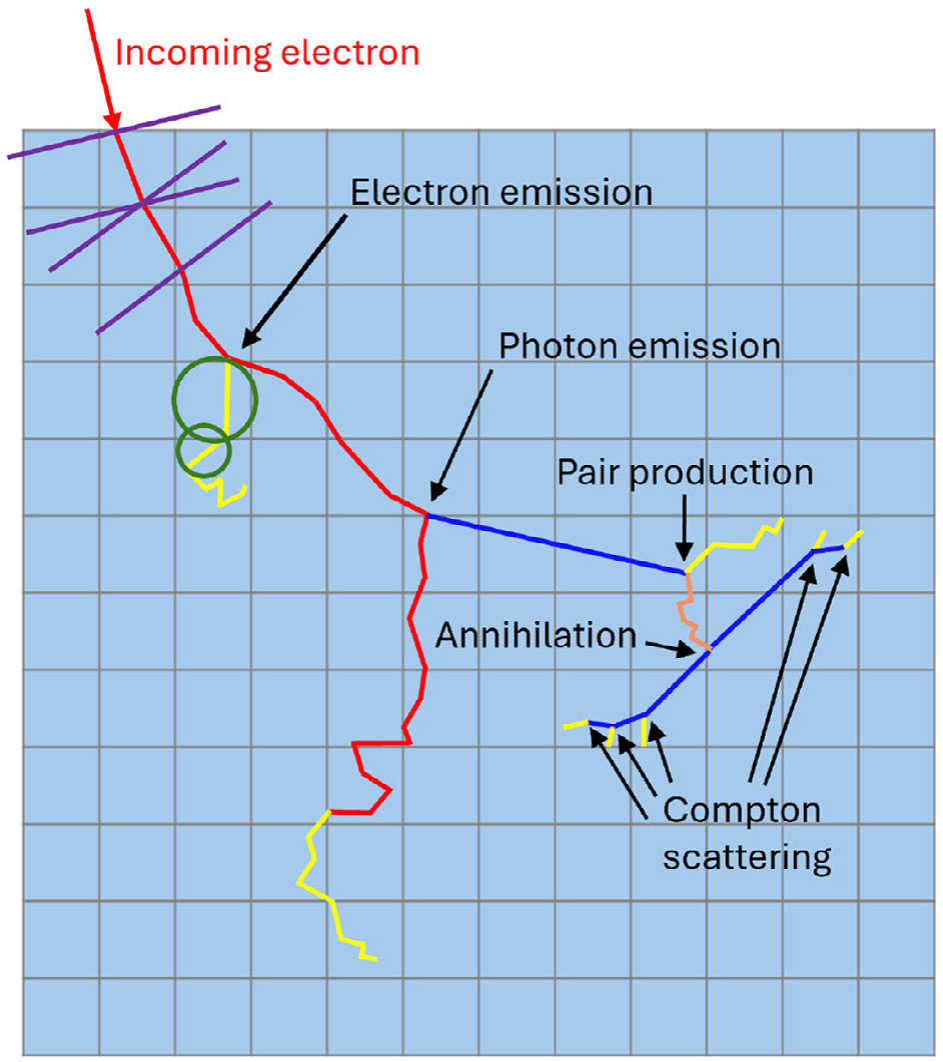
Schematic representation of the global simulation for an incoming electron traversing a voxelized absorber. The electron path (red) is modeled using slab‐based macro steps (purple): an initial slab step is followed by energy deposition via ray tracing, after which a new slab step begins at the exit point with orientation based on the direction. Whenever the electron energy falls below 25 MeV (yellow), spherical geometries are used for subsequent macro steps (green), with progressively smaller radii at lower energies. Photon and positron paths are represented in blue and orange, respectively.

In the global simulation, electrons are transported through the geometry in a stepwise manner using macro steps (Figure 2). The scored data obtained from the local simulations are converted into probability density functions (PDFs) and stored in a database.

For each macro step, the interaction channel is sampled. If the primary channel is selected, the energy loss, lateral displacement, and angular deflection are sampled from the corresponding PDFs. Energy deposition along the step is calculated using ray tracing between the entrance and exit points of the slab and scored in the dose grid. The electron energy and direction are then updated, and the next macro step is initiated. When the electron energy does not exactly match the discrete energies available in the database, linear interpolation between neighboring PDFs is applied.

If an electron emission or photon emission channel is sampled, the interaction location within the slab and the energy and direction of the secondary particle are sampled according to the PDFs stored in the database. The macro step is then terminated at the interaction location. The energy deposition between the slab entrance and interaction point is calculated as a fraction of the energy deposition of a full macro step, as obtained from the primary channel, with the fraction determined by the relative path length to the interaction point using the ray tracing procedure described previously. In the case of the electron emission channel, each of the two electrons subsequently initiates a new macro step. In the photon emission channel, the electron continues with a new macro step following the Bremsstrahlung interaction, while the photon is transported as described below.

**FIGURE 3:**
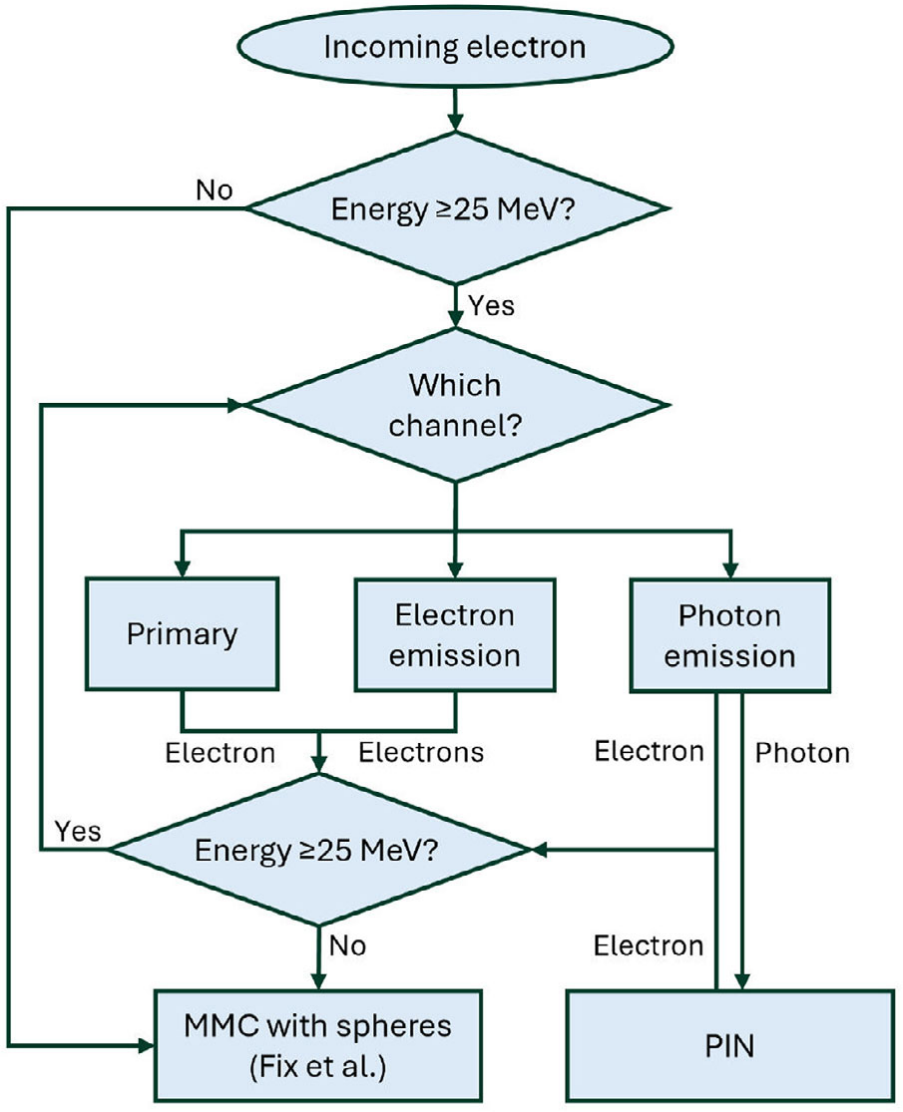
Schematic overview of particle transport in the MMC framework. The incoming electron is transported using slab‐based macro steps for energies ≥25 MeV; below this threshold, spherical macro steps (Fix et al.^23^, ^24^) are used. For slab‐based transport, channel‐dependent sampling of physical quantities is performed (primary, electron emission, or photon emission channel) and slab transport continues until the electron energy falls below 25 MeV, after which spherical macro steps are applied (Fix et al.^23^, ^24^). Photons produced in the photon emission channel are transported using PIN, and any electrons generated are reintroduced into the MMC transport loop. Positron transport is handled within the PIN framework.

Photons in the MMC framework are transported using an in‐house MC code modeling photon interactions (PIN),^27^, ^28^ which models Compton scattering, the photoelectric effect, and pair production based on XCOM cross‐section data. Positrons produced by pair production undergo Bhabha scattering and annihilation, which are also handled within PIN. All electrons are transported using slab‐based macro steps until their energy falls below 25 MeV. Below this threshold, the particle transport of the low energy electrons uses the existing transport model and precomputed data from Fix et al.^23^, ^24^ An overview of the MMC particle transport framework is shown in Figure 3.

To account for patient materials not explicitly included in the database, mixed materials are constructed by combining neighboring database materials according to their densities. This approach handles missing materials using the available database, avoiding the need to introduce a large number of additional materials to cover the nearly continuous range of densities present in patient CT data, while still enabling a continuous representation of patient heterogeneities. The mixing weights are determined by linear interpolation between the two nearest density values. For example, a density located midway between two database materials results in a 50% probability of sampling macro step characteristics from each material. MMC transport in patient geometries is then performed using these mixed materials without modification of the transport scheme.

### 2.3 MMC for VHEE validation

Unless stated otherwise, the following general settings were used throughout this study. All validation simulations employed monoenergetic parallel electron beams with energies from 50 to 250 MeV in 50 MeV increments. Dose distributions were scored on a 3D grid with a voxel resolution of 1 × 1 × 1 mm^3^. All phantoms (excluding patient cases) had dimensions of 40 × 40 × 40 cm^3^, with the beam incident along the central axis of the phantom. Statistical uncertainties were below 1% for all dose calculations. Comparisons between MMC and EGSnrc dose distributions were conducted using 3D gamma analysis with 2%/1 mm and 2%/2 mm (global) criteria and a dose threshold of 10% of the near‐maximum dose, defined as the mean dose of the 10 highest‐dose voxels.

**FIGURE 4:**
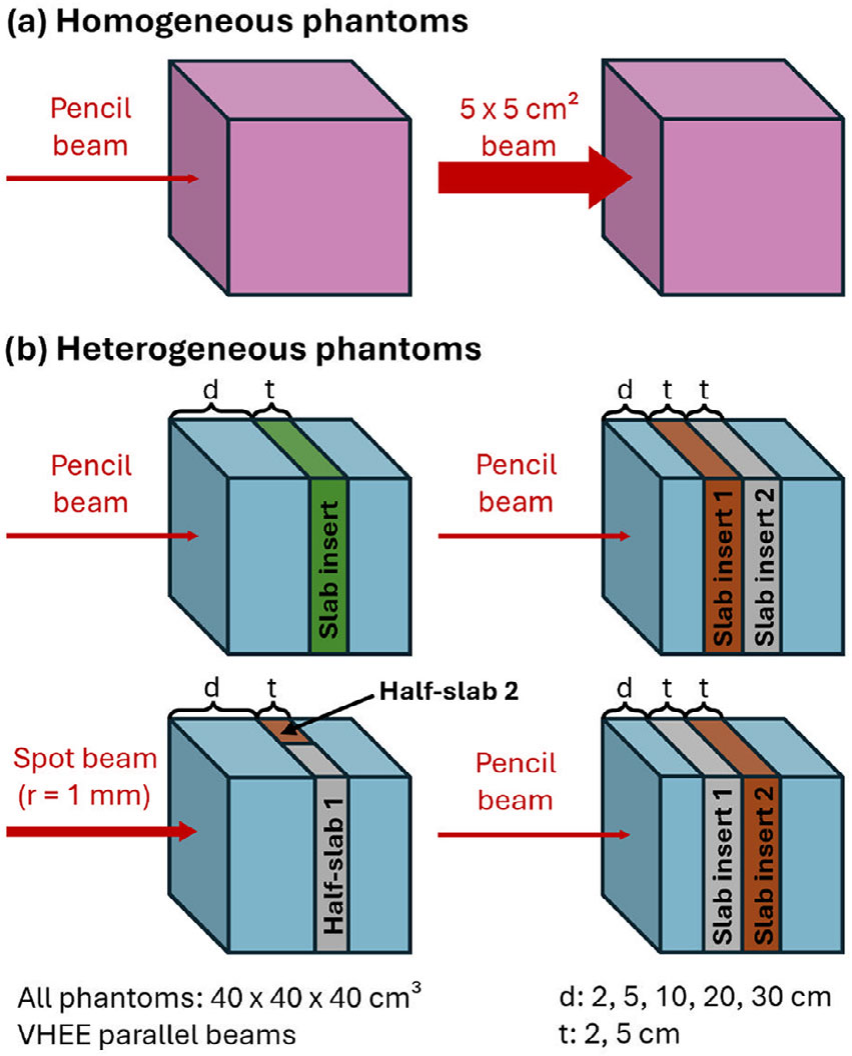
Phantoms used for validation of dose calculation in (a) homogeneous and (b) heterogeneous phantoms. Heterogeneous slab phantoms are obtained by inserting a lung and/or bone slab into a water phantom. Pink denotes an arbitrary material from the database, green denotes a generic slab that can represent either lung or bone depending on the configuration, while blue denotes water, and brown and gray denote lung and bone, respectively. d = depth in water to the heterogeneity (slab insert); t = slab insert thickness.

Validation was first performed in homogeneous phantoms composed of the specific materials included in the MMC database (Figure 4a). Monoenergetic electron pencil beams were simulated impinging perpendicularly on the center of the phantom, and MMC and EGSnrc dose distributions were compared using the evaluation criteria described above.

To validate mixtures of materials, homogeneous phantoms composed of mixtures containing 25%‐75% of adjacent database materials were simulated using the same beam configurations and evaluation settings. In addition to pencil beams, all simulations for homogeneous and mixtures of materials were repeated using square beams with a field size of 5 × 5 cm^2^.

Dose calculation in heterogeneous geometries was then assessed using slab phantoms in which one or two slabs of lung and/or bone material were embedded within a water phantom (Figure 4b). Monoenergetic pencil beams were simulated with a direction perpendicular to the material interfaces. Additionally, configurations with two adjacent half‐slabs of different materials placed at the same depth in water were investigated using spot beams with a radius of 1 mm directed through the lateral material interface (Figure 4b). Slab thicknesses of 2 and 5 cm were considered at depths of 2, 5, 10, 20, and 30 cm, allowing assessment of MMC performance across a wide range of longitudinal and lateral heterogeneities. Dose distributions were compared using the same gamma analysis criteria defined above.

Finally, dose calculations were performed for two patient CT datasets, a head‐and‐neck and a prostate case (Figure S1), selected to probe the MMC algorithm in the presence of strong tissue heterogeneities and over large transport distances. The head‐and‐neck case includes pronounced heterogeneities, such as air cavities and bone interfaces, while the prostate case represents a deep‐seated target with a large calculation volume. Monoenergetic square beams (5 × 5 cm^2^) were simulated, impinging on the left cheek and traversing the nasal cavity (head‐and‐neck case) or through the left hip and femur (prostate case). The same 5 × 5 cm^2^ field size used in the above validations was applied as this study does not yet consider realistic treatment plans. MMC and EGSnrc dose distributions were computed using the same gamma criteria described previously.

Computation times were recorded for all square beam simulations. The efficiency, ε, was defined as in Equation (1):

(1) $$\varepsilon =\frac{1}{T\sigma^{2}}\;$$

where T was the computation time and σ was the mean of the statistical uncertainties of all dose values with at least 50% of the maximum dose. Efficiency gains were quantified as the ratio of MMC efficiency to EGSnrc efficiency. All simulations were performed on an AMD EPYC system (2.25 GHz) equipped with 2 × 64 CPUs.

### 2.4 Neutron contribution

The potential neutron contribution to patient dose was assessed in simulations using Geant4 with the Penelope physics option. Monoenergetic electron pencil beams with energies ranging from 20 to 250 MeV in steps of 10 MeV were simulated impinging perpendicularly on the center of: (1) homogeneous phantoms composed of water, bone, or lung; (2) heterogeneous phantoms consisting of water with 10 cm thick lung or bone slab at 5 cm depth; and (3) heterogeneous phantoms split half by water and half by lung or bone, creating a material interface along the beam axis. All phantoms had a voxel resolution of 2 × 2 × 2 mm^3^. In addition, a more clinically relevant assessment was performed using two patient CT datasets, in which a 5 × 5 cm^2^ beam was simulated traversing the sternum or the pelvic region. These cases were selected because neutron production is more likely in high‐density materials, and both anatomical sites contain substantial bone structures. Simulations were performed both with and without hadronic interactions enabled (Supporting Material).

During the preparation of this manuscript, the authors used ChatGPT to improve clarity and language. All content was subsequently reviewed and edited by the authors, who take full responsibility for the final version of the publication.

## 3 RESULTS

The results of the MMC framework for VHEE validation are presented in this section and summarized in Table 1.

**TABLE 1:** Summary of gamma passing rates obtained from the MMC validation. Spot beams had a radius of 1 mm, while square beams had a field size of 5 × 5 cm^2^.

		Passing rates (%)	
Validation case	Beam shape	2%/1 mm	2%/2 mm
Homogeneous	Pencil	>99%	100%
	Square	>99%, except for air (>97%)	>99%
Heterogeneous	Pencil	>99%	>99%
	Spot	>99%	>99%
Head‐and‐neck case	Square	>94%	>97%
Prostate case	Square	>96%	>99%

**FIGURE 5:**
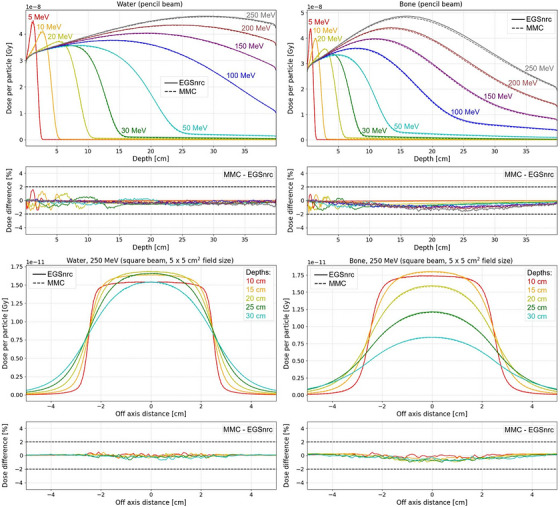
Top (pencil beam): Integrated depth dose curves for 5–250 MeV monoenergetic electron beams impinging on a water (left) or bone (right) phantom. Note that the results for energies below 50 MeV are included for illustrative purposes, to demonstrate the behavior and accuracy of the sphere‐based MMC transport; validation of the low‐energy MMC model has been comprehensively addressed in previous work.^23^, ^24^ Bottom (square beam, 5 × 5 cm^2^ field size): Lateral dose profiles at depths of 10, 15, 20, 25, and 30 cm for a 250 MeV monoenergetic electron beam impinging on a water (left) or bone (right) phantom.

Integrated depth dose (IDD) curves for monoenergetic electron pencil beams incident on a homogeneous water, bone, or lung phantom are shown in Figure 5 (top) and S2 (left). For bone, MMC calculations exhibit a lower dose at the distal fall‐off compared with EGSnrc, with the discrepancy increasing at higher beam energies (Figure 5, top right). Lateral dose profiles for a 250 MeV monoenergetic VHEE square beam with a field size of 5 × 5 cm^2^ incident on a water, bone, or lung phantom are presented in Figure 5 (bottom) and S2 (right). Overall, MMC dose distributions are in close agreement with the EGSnrc reference simulations, with differences remaining within 2% of the maximum dose across the investigated energy range. Corresponding 3D gamma analyses yielded passing rates exceeding 99% using the specified criteria for all materials, except for air with passing rates above 97% with a 2%/1 mm criteria. Mean dose differences, along with their corresponding standard deviations, for integrated depth dose curves and lateral dose profiles between MMC and EGSnrc simulations are summarized in Tables S1 and S2, respectively.

Similar agreement was observed for dose calculations in homogeneous phantoms composed of material mixtures. For all investigated mixture compositions, MMC and EGSnrc dose distributions showed consistent gamma passing rates above 99%, except for air mixtures with passing rates above 98% using 2%/1 mm criteria.

**FIGURE 6:**
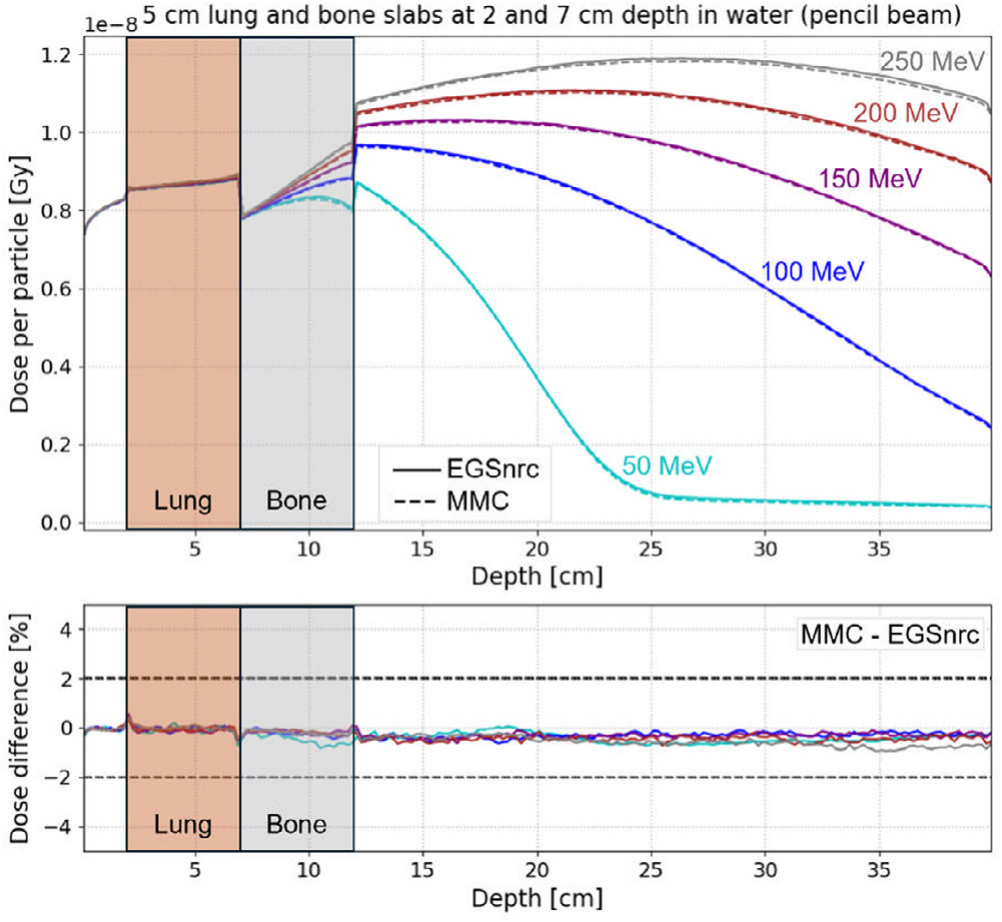
Integrated depth dose curves for 50–250 MeV monoenergetic electron pencil beams impinging on a slab phantom consisting of water with two 5 cm thick slab inserts of lung (brown) and bone (gray), located at depths of 2 and 7 cm in water, respectively.

For the heterogeneous slab phantoms, MMC reproduced the EGSnrc dose distributions with gamma passing rates exceeding 99% across all tested configurations. Figure 6 illustrates a representative IDD curve for a monoenergetic pencil beam traversing a heterogeneous slab phantom, demonstrating agreement within 2% between MMC and EGSnrc throughout the depth profile, including regions around material interfaces.

**FIGURE 7:**
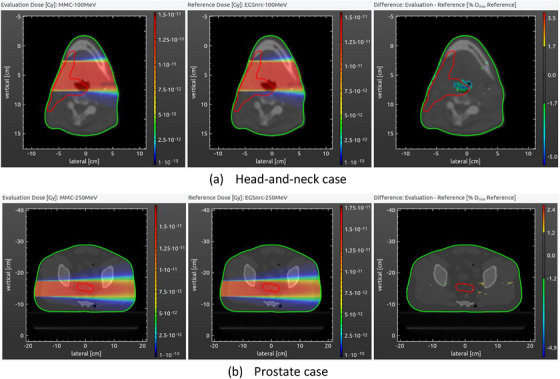
Dose distributions in an axial slice calculated with monoenergetic 5 × 5 cm^2^ parallel electron beams using MMC (left) and EGSnrc (middle), with the corresponding dose difference map shown on the right. (a) Head‐and‐neck case with a 100 MeV beam. (b) Prostate case with a 250 MeV beam. The planning target volume (PTV) and the body are contoured with red and green lines, respectively.

Dose distributions calculated for the head‐and‐neck and prostate patient CT datasets are shown in Figure 7. For the head‐and‐neck case (Figure 7a), larger differences were observed in the air cavities (5.1%) compared to other tissue materials (1.6%), based on the 99^th^ percentile of the dose difference between MMC and EGSnrc (normalized to the maximum dose). For both cases, MMC and EGSnrc dose calculations exhibited good agreement, with 3D gamma passing rates of 97% (head‐and‐neck case) and 99% (prostate case) using 2%/2 mm criteria.

The computational efficiency of MMC relative to EGSnrc is summarized as follows. The largest efficiency gains were observed for low‐density materials, particularly lung tissue, where MMC achieved speedups of up to 27 times compared with EGSnrc. For patient CT simulations, the efficiency gains were reduced compared with gains in homogeneous materials, reaching up to 11 times for the head‐and‐neck case and up to 12 times for the prostate case; nevertheless, MMC consistently provided an overall improvement of approximately one order of magnitude in computational efficiency. Absolute computation times are summarized in Table S3.

Geant4 simulations using the Penelope physics option, performed with and without hadronic interactions enabled, showed IDD differences within 0.3% for slab phantoms consisting of water, bone, and lung materials and total dose differences below 0.1% for the patient CT datasets (see Supporting Material). These results indicate that neutron contributions are negligible for the investigated VHEE energies and geometries and justify their exclusion from the MMC dose calculations.

## 4 DISCUSSION

In this study, an MMC method for VHEE dose calculation in radiotherapy was developed and validated. To the best of our knowledge, this represents the first MMC framework specifically applied to VHEE dose calculations. The use of both slab‐ and sphere‐based local geometries allows the method to reconstruct the predominantly forward‐directed transport of higher energy electrons, as well as the increased multiple scattering dominated regime at lower electron energies.

Another approach for VHEE dose calculation has previously been reported. In particular, Ronga et al. proposed an analytical dose calculation method based on the Fermi‐Eyges theory,^15^ achieving gamma passing rates above 90% using 2%/2 mm 2D gamma analysis in homogeneous water phantom and heterogeneous slab phantoms containing 5 cm thick bone or lung inserts. The method was later applied to a brain and a prostate case, although further improvements in accuracy were reported to be necessary.^16^ While analytical models provide fast dose calculations, they are limited in accurately modeling complex geometries and material compositions.

In contrast, the MMC approach presented here reproduces EGSnrc reference dose calculations within 2% agreement even in complex, heterogeneous scenarios, while providing an efficiency improvement of approximately one order of magnitude. Such gains in computational efficiency enable larger‐scale in‐silico investigations and facilitate the exploration of advanced VHEE treatment concepts.

Some differences observed in the results merit further discussion. In homogeneous bone phantoms, the MMC‐based IDD curves exhibit a lower dose at the distal fall‐off compared with EGSnrc, with the discrepancy increasing at higher energies (Figure 5, top right). This behavior is probably due to the simplified transport of charged particles within the PIN framework. While electrons are transported using MMC, positrons generated through photon‐induced pair production are handled within PIN and propagated along straight trajectories. As a consequence, positron track lengths, and thus their associated energy loss, are underestimated, particularly in high‐density, high‐Z materials. Since pair production increases with both energy and atomic number, the effect becomes more pronounced at higher energies and in bone. To address this limitation, future work will focus on improving positron transport modeling within the PIN framework or incorporating dedicated MMC positron data generated using EGSnrc. It should be noted, however, that the investigated case (40 × 40 × 40 cm^3^ bone phantom) corresponds to an extreme, non‐clinical scenario. Still, an agreement within 2% was achieved between MMC and EGSnrc.

In patient dose calculations, the largest discrepancies were observed in the head‐and‐neck case, predominantly within the air cavities (Figure 7a). This region is characterized by air‐like material, for which the MMC dose calculation shows reduced accuracy compared with denser tissues. This behavior is consistent with the validation results in homogeneous phantoms, where air exhibited lower gamma passing rates than other materials. However, because dose deposited in air is not clinically relevant for patient dose assessment, these discrepancies have limited impact on treatment evaluation. Nevertheless, future work could further improve dose accuracy in air through refined modeling of air‐specific particle transport.

The efficiency gains achieved with MMC were material dependent and were found to be highest in lung. This behavior can be understood by noting that efficiency is dependent on the number of transport steps. In low‐density materials, particles more frequently follow the primary channel, resulting in straighter trajectories with fewer interactions, fewer secondary particles, and consequently fewer macro steps than in higher density materials. However, the magnitude of the efficiency gain depends on the relative difference in step counts between MMC and EGSnrc. While air has a very low density, the highest efficiency gains were not observed in this medium because the difference in transport steps between the two methods is relatively small. For 50–250 MeV VHEE beams, MMC required on average 426 steps per particle in lung compared to 8972 steps for EGSnrc, whereas in air MMC required 401 steps compared to 756 steps for EGSnrc. As a result, the reduction in step count, and thus the efficiency gain, is much more pronounced in lung than in air. Future work could investigate the use of larger slab thicknesses for air to further improve computational efficiency while preserving acceptable accuracy.

This work has the following limitations. The validation phantoms were limited to dimensions of 40 × 40 × 40 cm^3^, which are insufficient to capture the full depth‐dose profiles for low density materials at the highest investigated energies, where electron ranges can exceed the phantom size. However, patient geometries are substantially smaller than those ranges, and the selected phantom size was sufficient for validating clinically relevant dose distributions while limiting computational cost. Should larger validation geometries be required in future studies, the same methodology can be readily extended.

Material mixtures in the MMC framework are generated using mass density interpolation between existing database materials, providing a practical means to represent the nearly continuous range of patient tissue densities in CT data. While this approach may introduce discrepancies for materials with similar densities but different elemental compositions, potentially affecting dose accuracy, the database could in principle be extended with additional materials. However, we have shown that a limited set of materials combined through interpolation is sufficient, as demonstrated by the good agreement between MMC and EGSnrc. Moreover, VHEE beams are known to be less sensitive to material heterogeneities than protons.^10^ As such, the current approach is considered adequate for patient dose calculations.

Neutron transport is not included in the present MMC implementation. This may lead to larger discrepancies in scenarios involving high‐density or high‐Z materials, such as metallic implants. However, Geant4 simulations indicate that neutron contributions are negligible for the investigated energies and materials, supporting their exclusion for patient dose calculations. If required, an additional neutron transport channel could be incorporated into the MMC framework. Moreover, the methodology is flexible and not tied to EGSnrc; other general‐purpose MC codes, such as Geant4, could be used for the local precalculations.

EGSnrc version 2020 was employed for all full general‐purpose MC simulations to maintain consistency with the work of Fix et al., who extensively validated MMC for lower energy electrons.^23^, ^24^

The current MMC implementation achieves efficiency gains of up to approximately 27‐fold compared with EGsnrc. Further improvements could be possible in future. For example, increasing the energy threshold used to classify low energy secondary particles could reduce the number of explicitly transported particles, at the expense of increased uncertainty in the spatial localization of energy deposition. This trade‐off may be acceptable for simulations employing larger voxel sizes, but its impact on dose accuracy requires further investigation. Additionally, the slab thickness used in the local simulations was fixed at 2 mm. Employing larger slab thicknesses could reduce the number of macro steps, provided that appropriate corrections for energy loss are applied. Finally, the adoption of an adaptive step size algorithm represents a promising avenue for further efficiency gains. Kueng et al. demonstrated that adaptive step sizing can substantially improve MMC efficiency for proton dose calculations while maintaining high accuracy.^26^

Overall, the proposed MMC framework provides accurate and efficient VHEE dose calculations in patient geometries and may support future research and clinical translation of VHEE‐based treatment modalities, including FLASH radiotherapy.

## 5 CONCLUSIONS

An MMC method for VHEE dose calculation was successfully developed and validated. The method enables accurate dose calculations in water and human tissue materials and achieves efficiency improvements of up to one order of magnitude compared with EGSnrc. Dose distributions calculated with MMC and EGSnrc showed good agreement within 2% for both head‐and‐neck and prostate patient CT cases, demonstrating the potential of MMC as an efficient and accurate tool for VHEE treatment planning studies.

## CONFLICT OF INTEREST STATEMENT

In his role as deputy editor for Medical Physics, author Michael K. Fix was blinded to the review process and had no role in decisions pertaining to this manuscript.

## Supporting information




**Supporting Information**: mp70539‐sup‐0001‐SuppMat.doc
