# Supplementary material for: Macro Monte Carlo dose calculation for very high energy electron (VHEE) radiotherapy
Source: Med Phys. 2026 Jun 22;53(7):e70539. doi: 10.1002/mp.70539 (PMC13285896; doi:10.1002/mp.70539)
Supplement: Supplementary file 1 — Supporting Information: mp70539‐sup‐0001‐SuppMat.doc [file MP-53-0-s001.doc]

**Supporting Material**


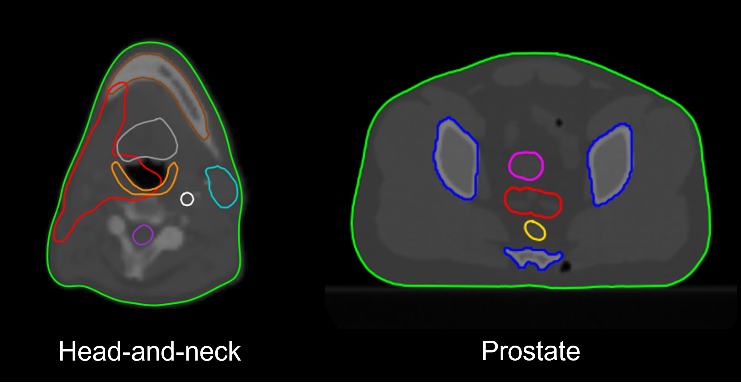


*Fig. S1 Left: Head-and-neck patient CT dataset showing contoured structures, including body (green), PTV (red), mandible (brown), oral cavity (gray), pharynx (orange), contralateral parotid gland (cyan), contralateral carotid artery (white), and spinal cord (purple). Right: Prostate patient CT dataset with contoured structures, including body (green), PTV (red), bladder (pink), rectum (yellow), and bones (blue).*

| 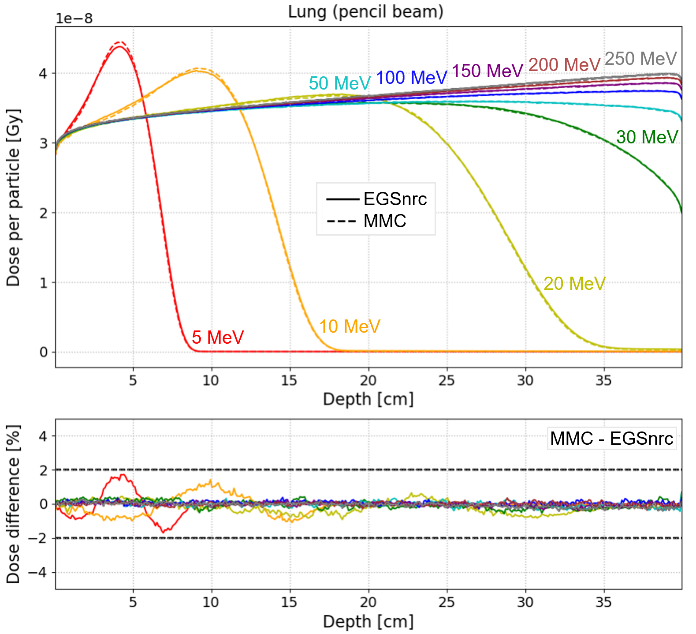 | 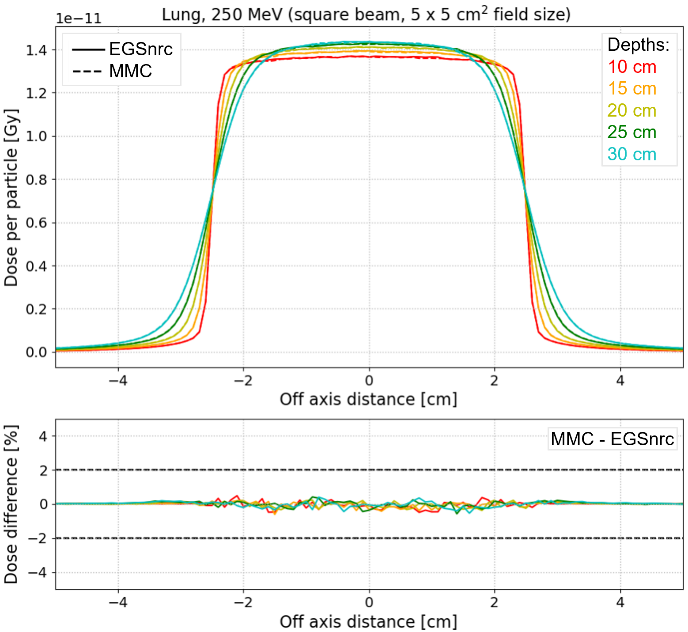 |
| --- | --- |

*Fig. S2 Left (pencil beam): Integrated depth dose curves for 5-250 MeV monoenergetic electron beams impinging on a lung phantom. Note that the results for energies below 50 MeV are included for illustrative purposes, to demonstrate the behavior and accuracy of the sphere-based MMC transport; validation of the low-energy MMC model has been comprehensively addressed in previous work^23,24^. Right (square beam, 5 x 5 cm^2^ field size): Lateral dose profiles at depths of 10, 15, 20, 25, and 30 cm for a 250 MeV monoenergetic electron beam impinging on a lung phantom.*

Mean dose differences, along with their corresponding standard deviations, for integrated depth dose curves between MMC and EGSnrc simulations using monoenergetic pencil beams are presented in Table S1:

*Table S1 Mean dose differences for integrated depth dose curves between MMC and EGSnrc for 50-250 MeV monoenergetic electron pencil beams impinging on homogeneous and heterogeneous phantoms.*

| *Phantom* | *Mean dose difference [%]* | | | | |
| --- | --- | --- | --- | --- | --- |
|  | *50 MeV* | *100 MeV* | *150 MeV* | *200 MeV* | *250 MeV* |
| *Air* | *-0.65 ± 0.53* | *-1.12 ± 0.54* | *-1.35 ± 0.51* | *-1.54 ± 0.56* | *-1.63 ± 0.50* |
| *Lung* | *-0.03 ± 0.14* | *0.05 ± 0.11* | *-0.03 ± 0.11* | *-0.01 ± 0.10* | *-0.07 ± 0.14* |
| *Water* | *-0.33 ± 0.25* | *-0.20 ± 0.15* | *-0.27 ± 0.16* | *-0.23 ± 0.15* | *-0.40 ± 0.21* |
| *PMMA* | *-0.36 ± 0.21* | *-0.25 ± 0.15* | *-0.20 ± 0.12* | *-0.30 ± 0.15* | *-0.35 ± 0.22* |
| *Bone* | *-0.56 ± 0.23* | *-0.65 ± 0.24* | *-0.69 ± 0.30* | *-0.72 ± 0.33* | *-0.86 ± 0.42* |
| *5 cm lung-bone inserts in water* | *-0.37 ± 0.23* | *-0.26 ± 0.16* | *-0.26 ± 0.14* | *-0.36 ± 0.19* | *-0.43 ± 0.28* |
| *5 cm bone-lung inserts in water* | *-0.33 ± 0.26* | *-0.23 ± 0.18* | *-0.26 ± 0.17* | *-0.33 ± 0.17* | *-0.45 ± 0.20* |

Mean dose differences, together with their corresponding standard deviations, for lateral dose profiles at depths of 10-30 cm between MMC and EGSnrc simulations of a 250 MeV monoenergetic 5 x 5 cm^2^ beam are presented in Table S2. Note that lateral dose profiles shown in Fig. 5 (bottom) and S2 (right) display only the central region of the phantom where the beam is incident (± 5 cm off axis), while the full phantom extends to ± 20 cm.

*Table S2 Mean dose differences for lateral dose profiles between MMC and EGSnrc at depths of 10-30 cm for a 250 MeV monoenergetic electron beam with a 5 x 5 cm^2^ field size impinging on homogeneous phantoms.*

| *Phantom* | *Mean dose difference [%]* | | | | |
| --- | --- | --- | --- | --- | --- |
|  | *10 cm* | *15 cm* | *20 cm* | *25 cm* | *30 cm* |
| *Air* | *-0.14 ± 0.25* | *-0.14 ± 0.25* | *-0.14 ± 0.24* | *-0.14 ± 0.25* | *-0.14 ± 0.25* |
| *Lung* | *-0.01 ± 0.09* | *-0.01 ± 0.07* | *0.00 ± 0.07* | *0.00 ± 0.07* | *0.00 ± 0.08* |
| *Water* | *0.02 ± 0.07* | *0.02 ± 0.07* | *0.01 ± 0.07* | *0.00 ± 0.09* | *-0.01 ± 0.07* |
| *PMMA* | *0.02 ± 0.06* | *0.03 ± 0.09* | *0.03 ± 0.08* | *0.02 ± 0.07* | *0.03 ± 0.08* |
| *Bone* | *0.02 ± 0.09* | *0.00 ± 0.16* | *-0.02 ± 0.24* | *-0.02 ± 0.24* | *-0.01 ± 0.22* |

**Computation times**

Computation times for MMC and EGSnrc simulations of 50-250 MeV monoenergetic electron beams of a 5 x 5 cm^2^ field size incident on homogeneous phantoms and patient cases to achieve a statistical uncertainty of 1% are presented below (Table S3).

*Table S3 Computation times for MMC and EGSnrc simulations of a 5 x 5 cm^2^ monoenergetic electron beam (50-250 MeV) incident on homogeneous and heterogeneous patient phantoms to achieve a statistical uncertainty of 1%. Simulations were performed using one CPU core on an AMD EPYC system (2.25 GHz) equipped with 2 x 64 CPUs.*

| *Phantom* | *Computation time [h]* | |
| --- | --- | --- |
|  | *MMC* | *EGSnrc* |
| *Air^a^* | *21.0-27.5* | *70.6-75.6* |
| *Lung^a^* | *3.3-9.4* | *91.2-123.8* |
| *Water^a^* | *4.3-12.6* | *109.6-161.3* |
| *PMMA^a^* | *4.5-12.5* | *72.2-162.1* |
| *Bone^a^* | *3.6-13.3* | *52.8-161.3* |
| *Head-and-neck^b^* | *1.3-2.6* | *6.6-16.1* |
| *Prostate^b^* | *0.5-1.2* | *5.8-11.1* |

*^a^ 1 x 1 x 1 mm^3^ voxels; ^b^ 2 x 2 x 2 mm^3^ voxels.*

**Neutron contribution investigations**

Preliminary investigations were conducted to assess the neutron contribution from very high energy electron (VHEE) beams. Monoenergetic VHEE beams (20-250 MeV) were simulated using Geant4 (Penelope physics option) both with and without hadronic interactions, allowing the estimation of the dose contribution from hadronic processes relative to purely electromagnetic interactions. The study was conducted using academic phantoms composed of water, bone, and lung, as well as two patient CT datasets. Patient CT datasets containing high-density implants were not included at this stage, as MMC has not yet been validated for metallic materials and metal-induced CT artifacts introduce additional uncertainties that are beyond the scope of this dose calculation validation.


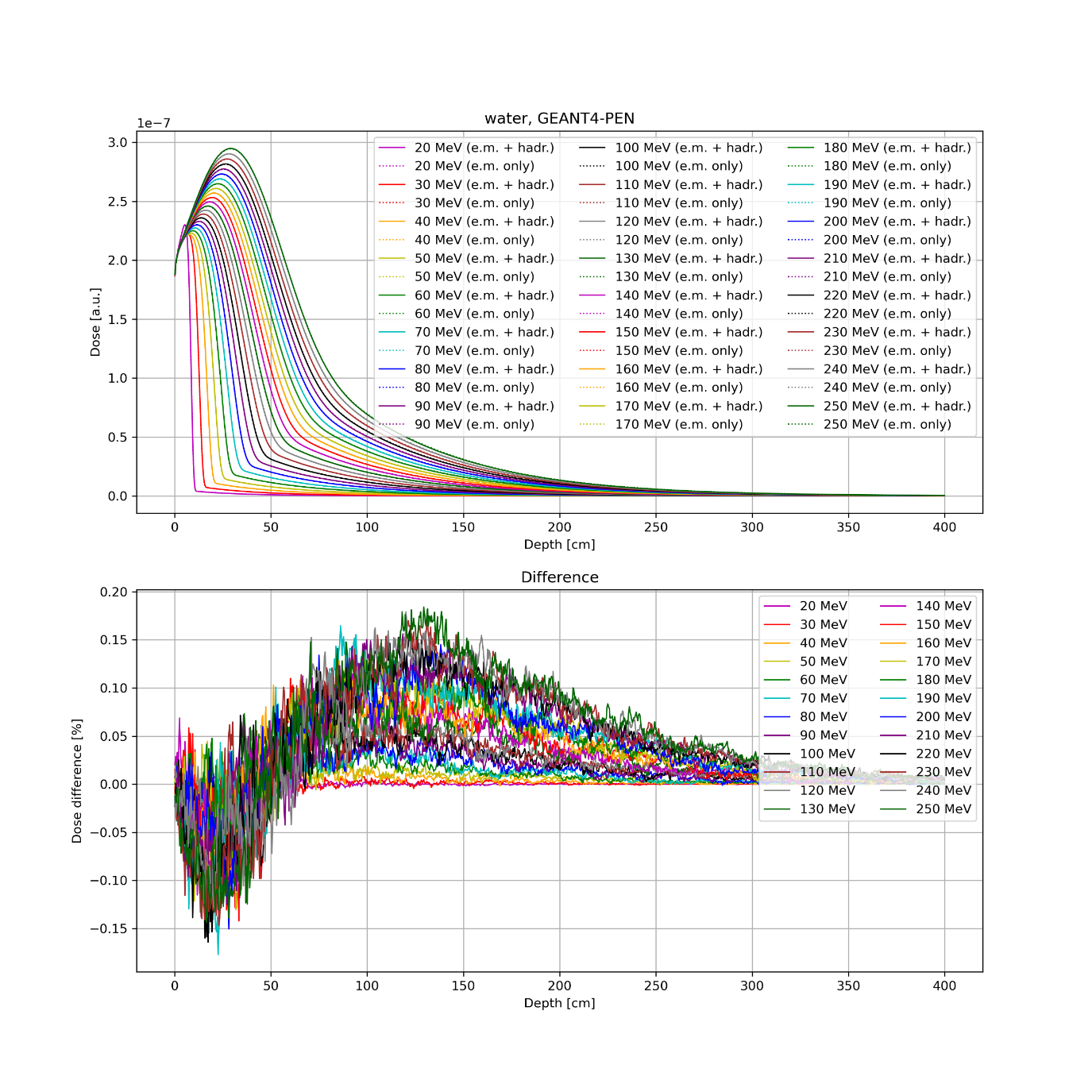


*Fig. S3 Integrated depth dose curves (top) and dose differences (bottom) for Geant4 simulations of monoenergetic electron pencil beams (20-250 MeV) impinging on a homogeneous water phantom performed with and without hadronic interactions.*


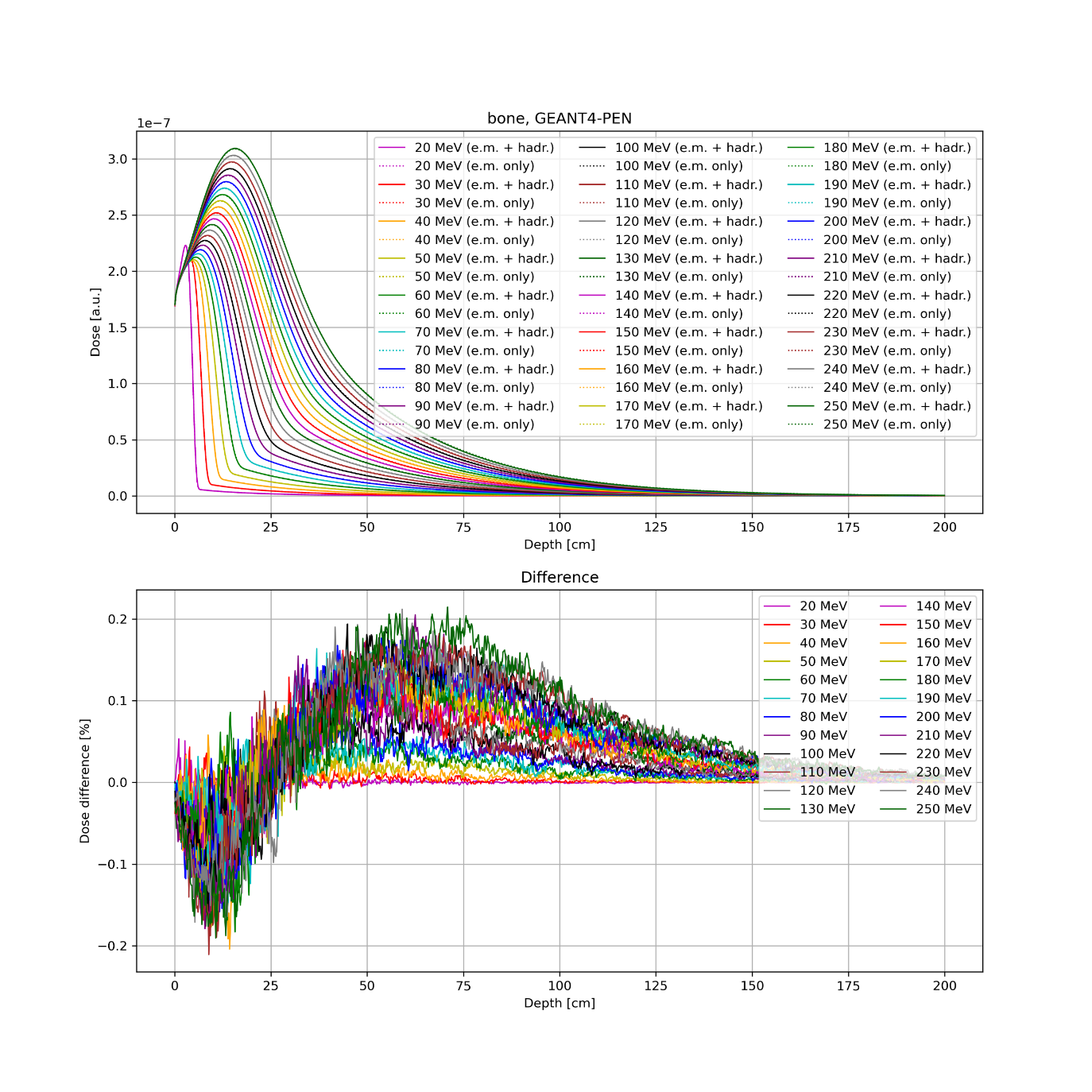


*Fig. S4 Integrated depth dose curves (top) and dose differences (bottom) for Geant4 simulations of monoenergetic electron pencil beams (20-250 MeV) impinging on a homogeneous bone phantom performed with and without hadronic interactions.*


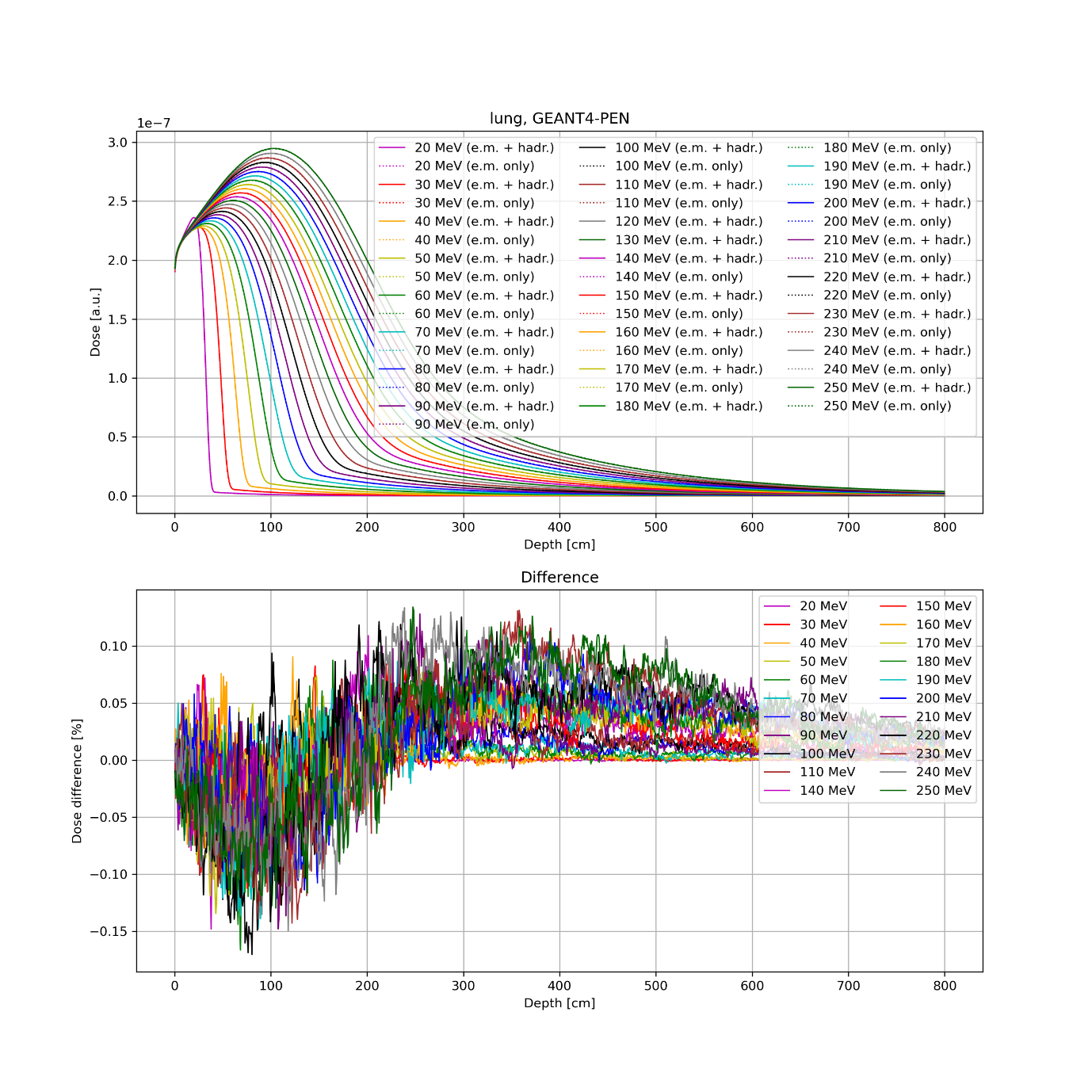


*Fig. S5 Integrated depth dose curves (top) and dose differences (bottom) for Geant4 simulations of monoenergetic electron pencil beams (20-250 MeV) impinging on a homogeneous lung phantom performed with and without hadronic interactions.*


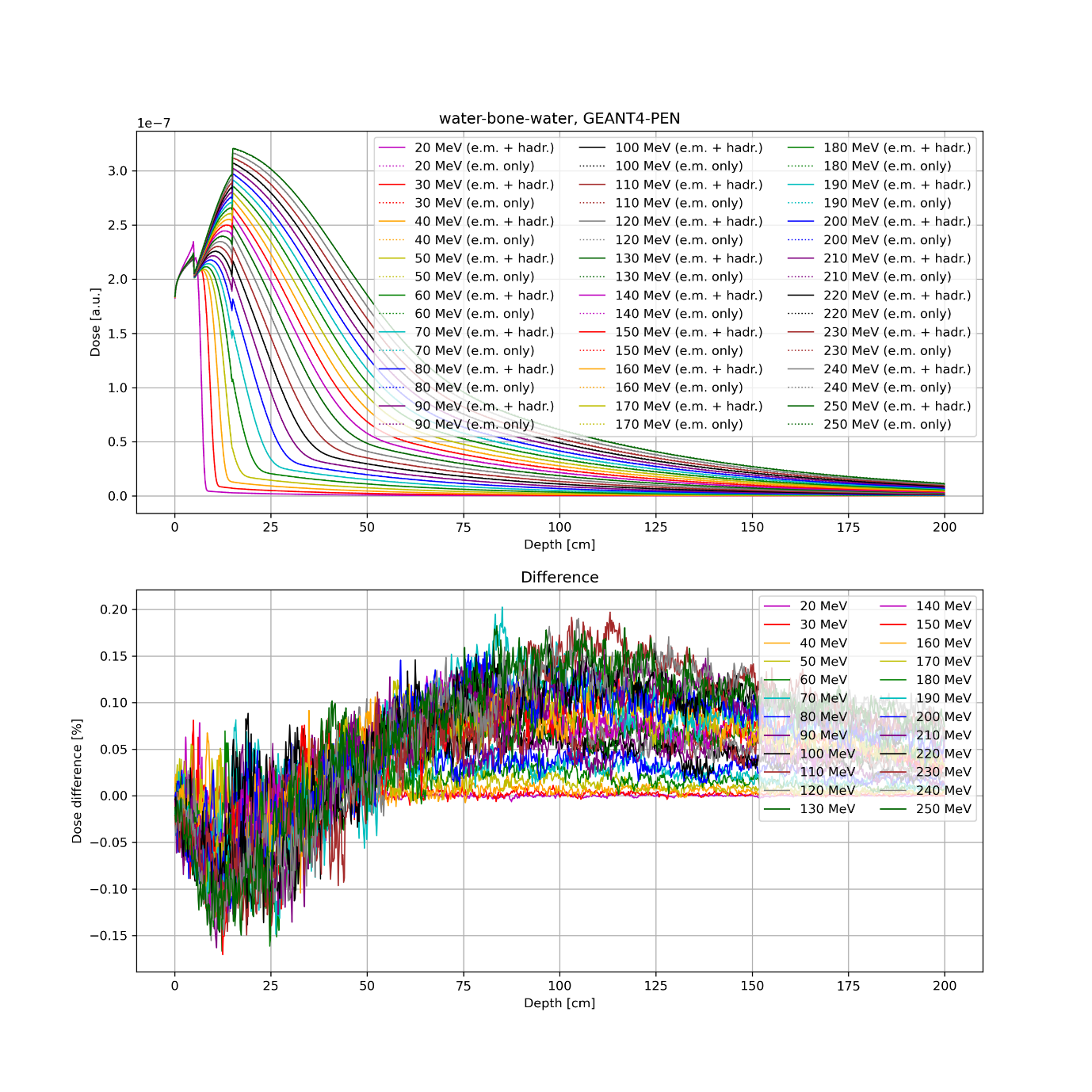


*Fig. S6 Integrated depth dose curves (top) and dose differences (bottom) for Geant4 simulations of monoenergetic electron pencil beams (20-250 MeV) impinging on a heterogeneous slab phantom performed with and without hadronic interactions. The phantom consists of water with a 10 cm thick bone insert.*


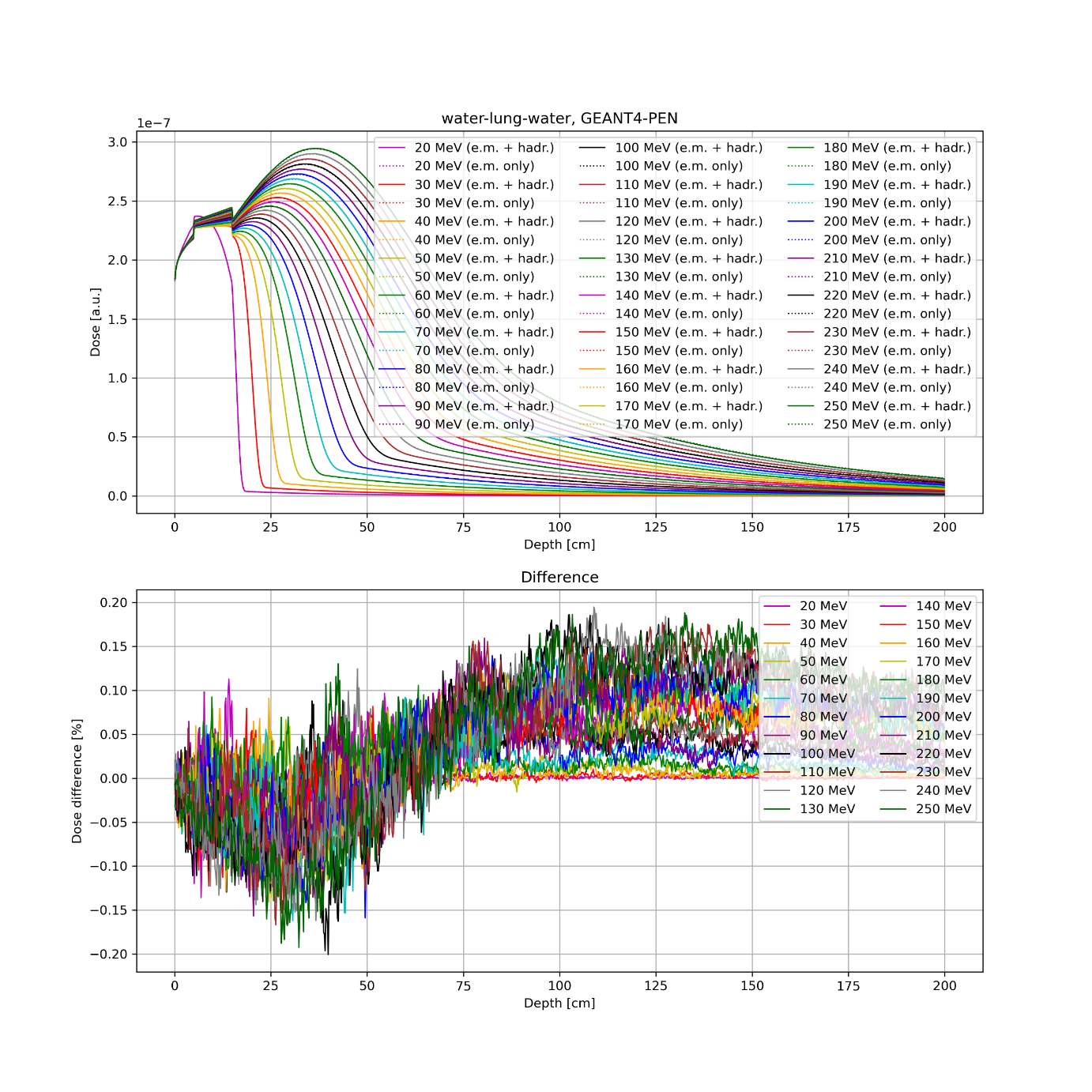


*Fig. S7 Integrated depth dose curves (top) and dose differences (bottom) for Geant4 simulations of monoenergetic electron pencil beams (20-250 MeV) impinging on a heterogeneous slab phantom performed with and without hadronic interactions. The phantom consists of water with a 10 cm thick lung insert.*


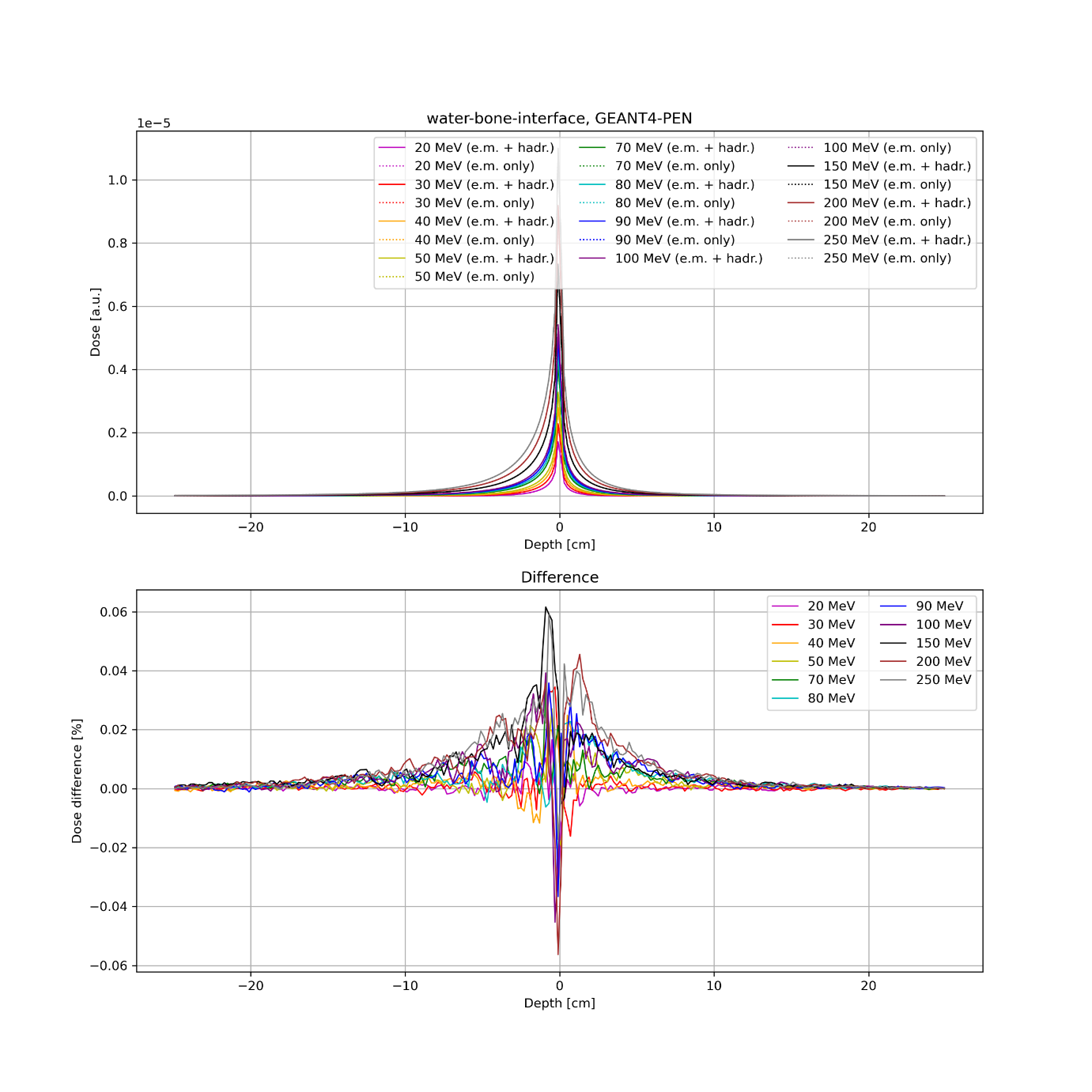


*Fig. S8 Integrated dose profiles (top) and dose differences (bottom) for Geant4 simulations of monoenergetic electron pencil beams (20-250 MeV) impinging on a heterogeneous phantom performed with and without hadronic interactions. The phantom is composed of water and bone, with the pencil beam passing through the material interface.*


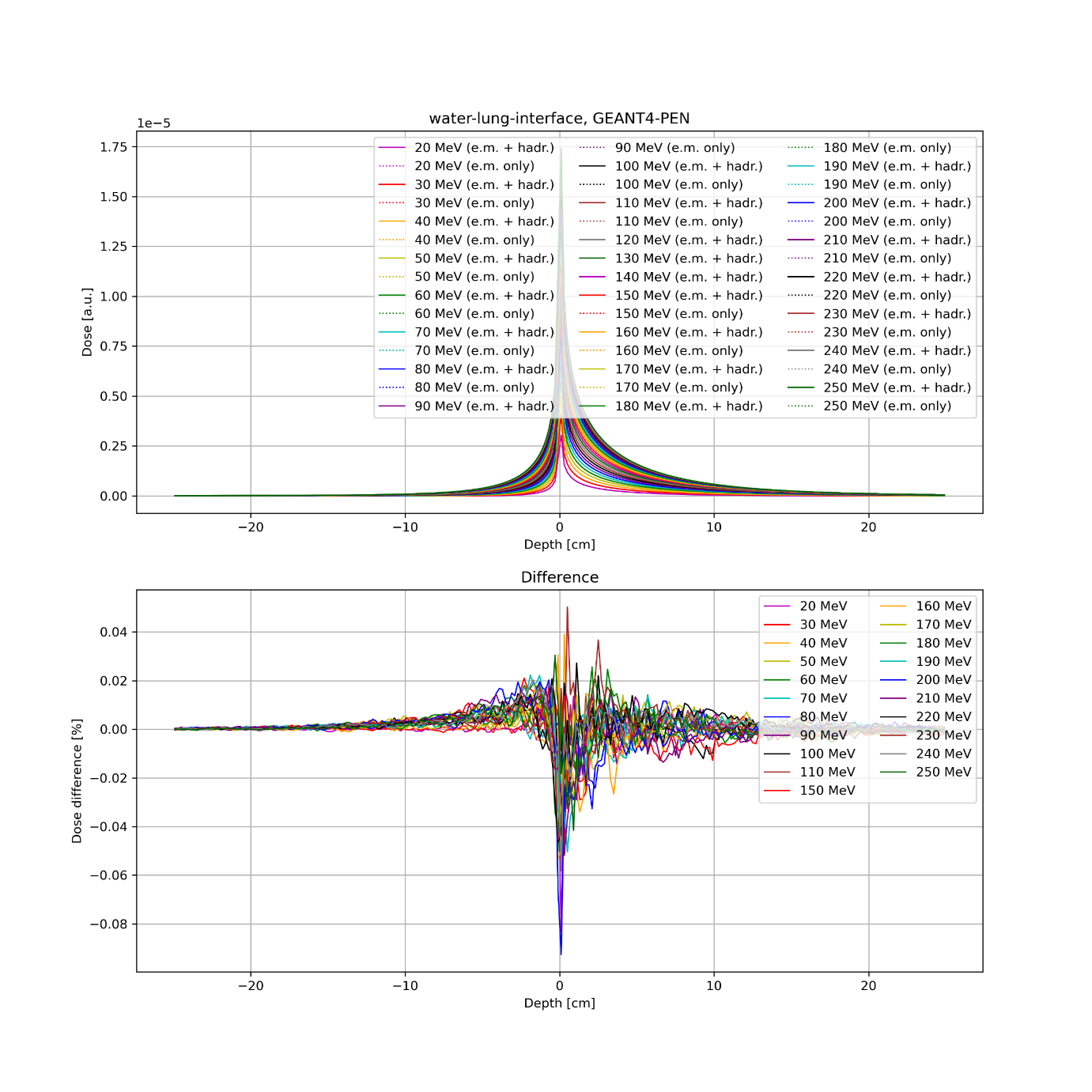


*Fig. S9 Integrated dose profiles (top) and dose differences (bottom) for Geant4 simulations of monoenergetic electron pencil beams (20-250 MeV) impinging on a heterogeneous phantom performed with and without hadronic interactions. The phantom is composed of water and lung, with the pencil beam passing through the material interface.*


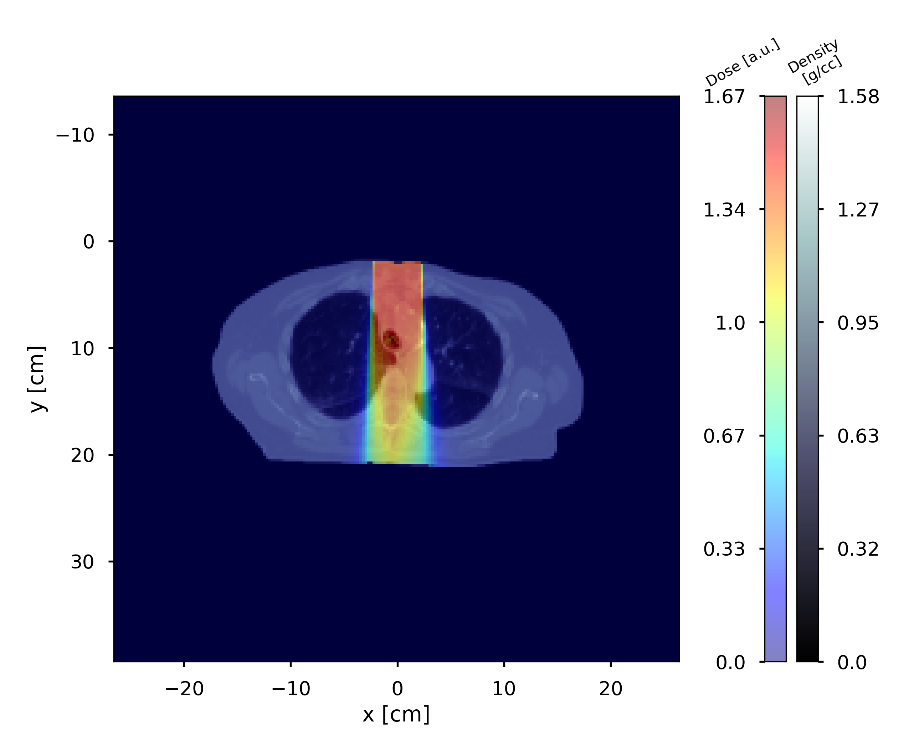


*Fig. S10 Example axial dose distribution of a 5 x 5 cm^2^ monoenergetic VHEE beam (100 MeV) incident on a lung patient case, with the beam traversing the sternum.*


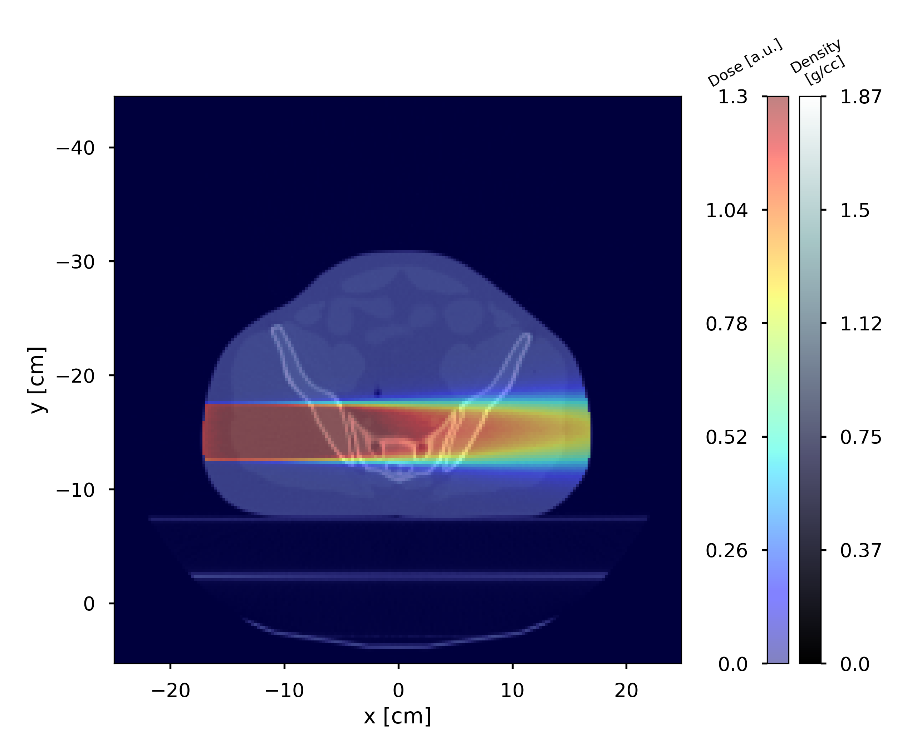


*Fig. S11 Example axial dose distribution of a 5 x 5 cm^2^ monoenergetic VHEE beam (250 MeV) incident on a prostate patient case, with the beam traversing the pelvic region.*

*Table S4 Total dose differences between Geant4 simulations of a 5 x 5 cm^2^ monoenergetic electron beam performed with and without hadronic interactions for a lung and a prostate patient case. Differences are normalized to the mean of all dose values exceeding 50% of the dose maximum.*

| *Energy [MeV]* | *Dose diff.* |  |
| --- | --- | --- |
|  | *Lung case* | *Prostate case* |
| *20* | *-0.00753%* | *-0.00066%* |
| *50* | *0.00594%* | *-0.01764%* |
| *100* | *-0.03865%* | *-0.03747%* |
| *150* | *-0.05901%* | *-0.06238%* |
| *200* | *-0.05218%* | *-0.06935%* |
| *250* | *-0.06248%* | *-0.09224%* |
